# Glucose promoting the early embryonic development by increasing the lipid synthesis at 2-cell stage

**DOI:** 10.3389/fcell.2023.1208501

**Published:** 2023-07-18

**Authors:** Mingwen Wang, Jinfeng Xue, Chanyi Li, Lingbin Qi, Lan Nie, Zhigang Xue

**Affiliations:** ^1^ Reproductive Medical Center, Tongji Hospital, Tongji University School of Medicine, Shanghai, China; ^2^ Department of Infertility and Endocrine, Maternal and Child Health Hospital of Hunan Province, Changsha, China

**Keywords:** embryo development, metabolite, culture media, glucose, lipid biosynthesis

## Abstract

The optimization of culture conditions is one of the main strategies to improve the embryo development competence in *in vitro* fertilization (IVF). Glucose is an important carbon source while also exists in the oviductal fluid *in vivo*, the effect of glucose in embryo development microenvironment is still unclear. Here we employed the LC-MS to detect and analyze the metabolites in the culture medium of different cleavage stages including 2-Cell, 4-Cell and 8-Cell mouse embryos, respectively. The effects of the external glucose were estimated by measuring the development rate at different glucose concentrations from 0 to 5 mmol/L, and the gene expression changes were detected to explore the potential mechanism after the addition of glucose in the media. Our results indicated the 2-Cell and 8-Cell stages had defined characteristic metabolites, while 4-Cell stage was the transition state. Global and contiguous metabolic characteristics showed the glycometabolism play a critical role at each early cleavage stages during the embryo development. The 8-Cell rates demonstrated the addition of glucose in culture media significantly improve the embryo competence, the highest rate was 87.33% using 3 mmol/L glucose in media, in contrast only 9.95% using the media without glucose. Meanwhile, the blocked embryos were mainly enriched at 2-Cell stage. Further transcriptome study found 3 mmol/L glucose in media remarkably upregulated the gene expression of lipid biosynthesis at 2-Cell stage, the increased lipid was confirmed by nile red staining. These data indicated the glucose may promote the development competence through increasing the lipid biosynthesis to overcoming the 2-Cell block. Our findings were helpful for the further optimization of IVF culture media, as well as the estimation of embryo quality using metabolites in the culture media.

## 1 Introduction

Embryonic early development undergoes multiple process including zygote genome activation (ZGA), remodeling of epigenetics, as well as dramatic changes of metabolism (Xue et al., 2013; Sharpley et al., 2021; Xu et al., 2021). Except the morphological screening of gametes, regulating the metabolism of embryos through culture conditions is the main strategy to improve the embryo development competence in *in vitro* fertilization (IVF) (Chronopoulou and Harper, 2015). In the past decades, various components have been tested and proven to be optimized for the embryonic culture media, including amino acids, sodium lactate, sodium pyruvate, serum et al., as well as the glucose, which role is still unclear during the embryonic cleavage stage (Chronopoulou and Harper, 2015).

The influence of glucose for the early development is controversial for decade years. Glucose was found to contribute to the 2-Cell block, elimination of glucose from modified TLP-PVA culture media supported embryos to overcome the block (Schini and Bavister, 1988; Chatot et al., 1989), but other studies showed the presence of glucose or fructose is essential for the early development (Ludwig et al., 2001). Recent study found glucose controlled the cell fate specification during morula stage to the inner cell mass and trophectoderm after 8-Cell stage (Chi et al., 2020), but its role in early cleavage stages is still unclear. Considering glucose is essential for the energy metabolism and nucleotide biosynthesis, lipid biosynthesis and redox homeostasis (Leone and Powell, 2020), while glucose is also present in the oviductal fluid including human, mouse and other species (Carlson et al., 1970; Lippes et al., 1972; Gardner and Leese, 1990; Nichol et al., 1992), effect of glucose in embryo development microenvironment needs to be further explored.

In the present study, we cultured the embryos in K+ simplex optimized medium (KSOM) (Lawitts and Biggers, 1992) supplemented with different concentrations of glucose, then the metabolites in the embryo culture medium from 2-Cell stage to 8-Cell stage were determined, and we further investigated the effects of different glucose concentrations in culture medium for the development rate and analyzed the gene expression changes caused by the addition of glucose at 2-Cell stage.

## 2 Materials and methods

### 2.1 Ethics statement

All experimental animals and reagents were supervised and allowed by the Animal Ethics Committee of the Tongji medical school University, Shanghai, China. All methods follow the stated guidelines and guidelines, unless there are special precautions. All animals used in this study were purchased from Viton Lever. Reagents were purchased from Aibei.

### 2.2 Fertilization and single embryo preparation

Adult female (8–10 weeks old) and adult male (10–12 weeks old) ICR mice were used as donors for oocytes and sperm. Pregnant mare’s serum gonadotropin (PMSG) 10 IU per animal was intraperitoneally injected, and human chorionic gonadotropin (hCG) 10 IU per animal was intraperitoneally injected 48 h later. Prepare the required Petri dishes a day in advance and equilibrated overnight. The male mice that had not mated within 3–5 days were sacrificed by neck break, the tail epididymis and vas ductus were excised, and their sperm was placed in TYH solution. The TYH dish was incubated in an incubator at 37°C and 5% CO_2_ for 1 h, so that sperm could obtain energy. The cumulus complex was incubated with sperm in an incubator of 5%CO_2_ at 37°C for 8 h.The zygotes were washed and transferred to KSOM solution for culture until 8-Cell stage. Each embryo is collected at a specific point in time, and one embryo is placed in a small tube.

### 2.3 Collection of embryo culture medium

After *in vitro* fertilization, embryo culture medium of 2-Cell stage, 4-Cell stage and 8-cell stage were collected for non-targeted metabolomics studies at 24, 48, and 72 h after fertilization, respectively. Ten embryos were cultured in about 30 µL per droplet. If 9 or more embryos meet the quality embryo morphology criteria, the droplet is included in the study. There were 6 replicates per developmental stage. Each sample is about 100 µL. Store in liquid nitrogen for later testing.

### 2.4 Non-targeted metabolomics

Melt all samples at 4°C. 100 μL from each sample was placed in a 2 mL centrifuge tube. Each centrifuge tube was added 400 µL methanol (−20 °C), shook for 60s, and thoroughly mixed. After centrifugation at 12,000 rpm for 10 min at 4°C, all the supernatant was taken and transferred to a new 2 mL centrifuge tube for vacuum concentration and drying.

150 µL 2-chlorophenylalanine (4 ppm) 80% methanol solution was redissolved, and the supernatant was filtered using 0.22 µm membrane to obtain the samples to be tested. Merchant detection, with Thermo Vanquish instrument, is automated using ACQUITY UPLC^®^ HSS T3 1.8 µm (2.1 mm × 150 mm) column.

The injector temperature was set at 8°C, the flow rate was 0.25 mL/min, the column temperature was 40°C, and the sample was 2 μL for gradient elution. Conditions of mass spectrum: Thermo Q Exactive Plus, electrospray ion source (ESI), positive and negative ion ionization mode, positive ion spray voltage of 3.50 kV, negative ion spray voltage of 2.50 kV, sheath gas of 30 arb, auxiliary gas of 10 arb. The capillary temperature was 325°C, the full sweep was performed at a resolution of 70,000, and the sweep range was 81–1,000. HCD was used for secondary cracking, the collision voltage was 30 eV, and the unnecessary MS/MS information was removed by dynamic exclusion.

### 2.5 Metabolic data analysis

After the LC-MS detaction, the peak intensity of each metabolite was batch normalized and input to MetaboAnalyst 5.0 webset for further analysis (Pang et al., 2021). The data was log transformed and scaled, then fold change between groups were calculated and samples were clustered using sparse PLS-DA, the abundance of the characteristic metabolites were showed using heat map and volcano map.

### 2.6 Library construction and single cell RNA sequencing

Embryos were collected (one embryo per sample) in a 0.2-mL PCR tube with a micro-capillary pipette and each sample was added with 1.2 μL lysis buffer (0.2% Triton X-100, Rnase inhibitor), then dNTPs, oligo-dT primers were added in sequence and immediately incubated at 72°C for 3 min, and processed into cDNA with Superscript II reverse transcriptase. The cDNA was amplified with KAPA Hifi HotStart using ∼17 cycles. Sequencing libraries were constructed from 0.1 ng of pre-amplified cDNA using DNA library preparation kit (TruePrep DNA Library Prep Kit V2 for Illumina, Vazyme). Libraries were sequenced on a HiSeq PE150, with paired-end reads of 100-bp long each.

### 2.7 RNA-seq data processing

For RNA-seq analysis of early stage embryos, FastQC was performed for Illumina reads. We used the Trim Galore software to discard low-quality reads, trim adaptor sequences, and eliminate poor-quality bases. Then, we downloaded the mouse reference genome (genome assembly: mm10) from the Ensembl database and used the HISAT2 software for read alignment. The gene-level quantification approach was used to aggregate raw counts of mapped reads using the featureCounts tool. The expression level of each gene was quantified in terms of the normalized fragments per kilobase of transcript per million mapped reads (FPKM). Next, we used the R package DESeq2 for differential gene expression analysis. KEGG analysis of screened DEGs was performed using the KOBAS online tool (http://kobas.cbi.pku.edu.cn/kobas3/).

### 2.8 Nile red staining

Fixation: The embryos of the control group and the hypoxic group were washed in different PBS solutions for 3 times, and then carefully transferred to 4% paraformaldehyde (sealing solution) and placed at room temperature for 30 min. The embryos were transferred to clean Petri dishes and washed three times in PBS drops. Penetration: 30 min at 0.13%TritonX-100 room temperature. Similarly, the embryos were transferred to clean Petri dishes and washed three times in PBS drops. Staining: The embryos were placed in Nile red stained drops, shielded from light for 30 min, cleaned in clean PBS drops, observed under an inverted microscope, and the exposure time was set at 3 s.

### 2.9 Statistical analysis

All statistical results are presented as the mean ± SD. The data were analyzed by one way -ANOVA. Statistical significance was assessed using GraphPad Prism software 9.0, and *p* < 0.05 indicated statistical significance.

## 3 Results

### 3.1 The global characteristics of metabolism during early cleavage

**FIGURE 1:**
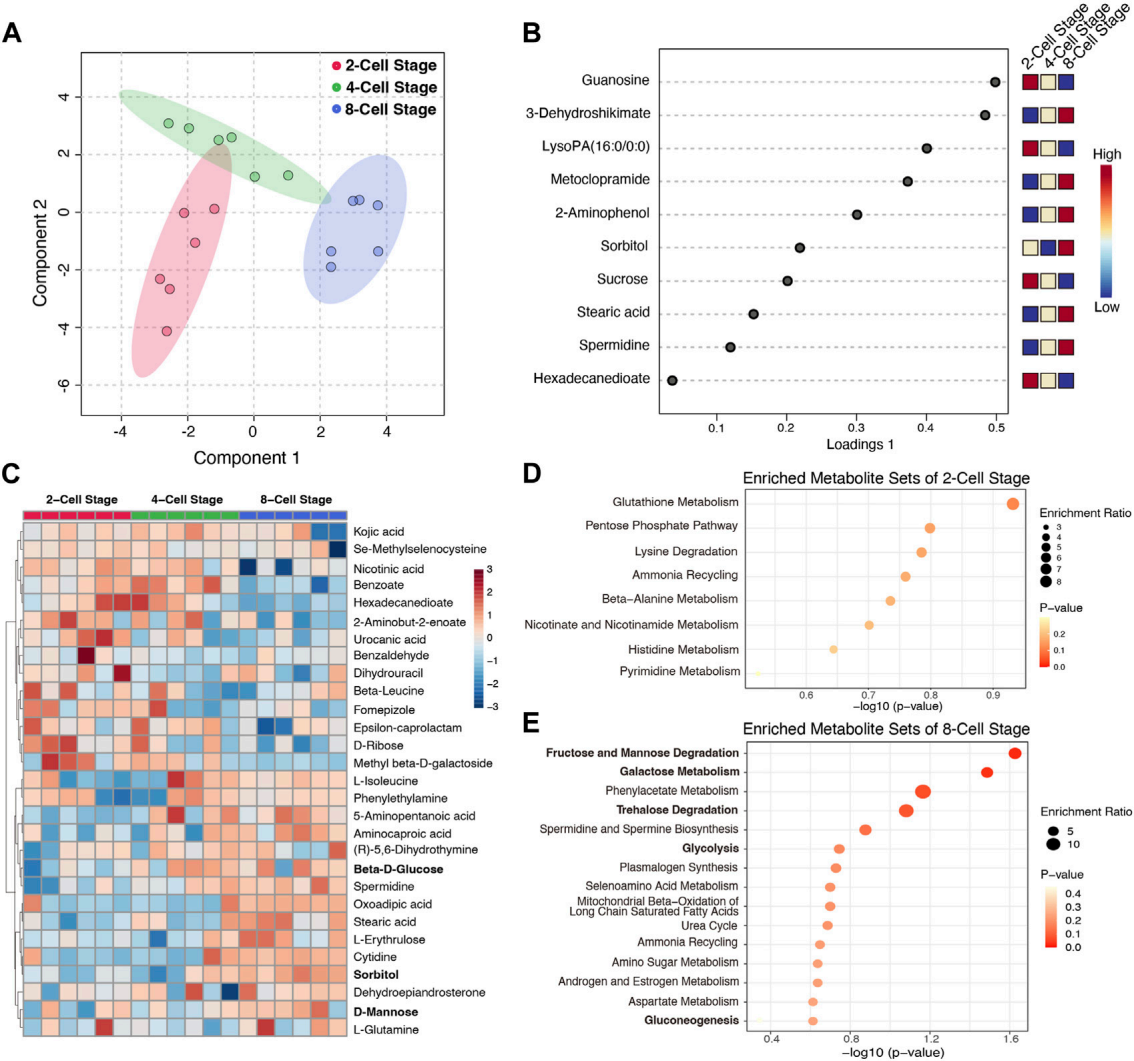
The metabolic characteristics of culture medium at different cleavage stages. **(A)** Sparse PLS-DA analysis using the detected metabolites in different culture medium; **(B)** The loadings plot of the top variable metabolites selected by the sparse PLS-DA model. The variables are ranked by the absolute values of their loadings; **(C)** The abundance heatmap of 31 characteristic metabolites at different stages; **(D,E)** The enriched metabolite sets of characteristic metabolites at 2-Cell and 8-Cell stages, respectively.

To determine the metabolic profile of the culture medium at each cleavage stage, we employed the sequential culture methods, the mouse zygotes were continuously cultured to the 8-Cell stage in KSOM medium, and we detected the metabolic characteristics in the culture medium from 2-Cell to 8-Cell stage using the non-target metabolomics techniques based on LC-MS. 120 metabolites were identified and the peak intensities were normalized for further analysis. The Sparse PLS-DA analysis showed different stage medium samples could be well distinguished by these metabolites (Figure 1A), while the loadings plot shows the top variable metabolites among stages were mainly enriched at the 2-Cell stage and 8-Cell stage (Figure 1B), suggesting after fertilization, the embryo secreted metabolites into the culture medium at each cleavage stage, while 4-Cell stage showed a transition state between 2-Cell stage and 8-Cell stage. Subsequently, we calculated the enriched metabolites at each stage compared with the other two stages based on fold changes, 15 metabolites, 3 metabolites and 13 metabolites were identified from the 2-Cell stage to 8-Cell stage, respectively (Supplementary Figure S1A). The abundances of these 31 characteristic metabolites were also mainly enriched at the 2-Cell stage and 8-Cell stage, and divisively enriched at the 4-Cell stage (Figure 1C; Supplementary Figure S1B), consistent with the top variable metabolites, suggesting the 2-Cell stage and 8-Cell stage have definite metabolic characteristics. Further enrichment analysis indicated characteristic metabolites of the 2-Cell stage were mainly involved in glutathione metabolism, pentose phosphate pathway (PPP), lysine degradation and others (Figure 1D), while characteristic metabolites of the 8-Cell stage were remarkably enriched in glycometabolism including fructose and mannose degradation, galactose metabolism, trehalose degradation, glycolysis and gluconeogenesis (Figure 1E), indicating the potentially critical role of glycometabolism during early cleavage.

### 3.2 The contiguous metabolic characteristics along with the early cleavage stages

**FIGURE 2:**
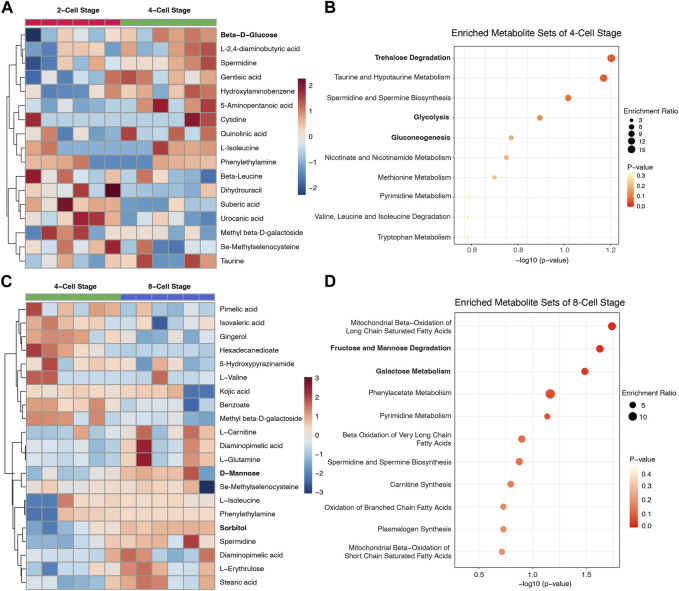
The differential metabolites of culture medium and enrichment analysis between contiguous stages. **(A)** The abundance heatmap of the differential metabolites between 4-Cell and 2-Cell stages; **(B)** The enriched metabolite sets of 4-Cell stage compared to 2-Cell stage; **(C)** The abundance heatmap of the differential metabolites between 8-Cell and 4-Cell stages; **(D)** The enriched metabolite sets of 8-Cell stage compared to 4-Cell sta Figure. 3 The effect of glucose on embryo development. **(A)** The embryo morphology cultured by different glucose concentrations at the 8-Cell stage; **(B)** The 8-Cell embryo rates of culture medium with different glucose concentrations; **(C)** The 2-Cell block embryos cultured by different glucose concentrations at the 4-Cell stage; **(D)** The 2-Cell block rates of culture medium with different glucose concentrations.

To further determine the dynamic metabolic changes and properties in culture media from 2-Cell to 8-Cell stage, we compared each stage with the contiguous stage individually. 10 metabolites including beta-D-glucose enriched in 4-Cell stage when compared to 2-Cell stage (Figure 2A; Supplementary Figure S2), and these components were involved in trehalose degradation, taurine and hypotaurine metabolism as well as glycolysis, gluconeogenesis (Figure 2B), reflecting the glycometabolism was also play an important role at 4-Cell stage. Subsequently, 11 metabolites including D-mannose and sorbitol were abundant in 8-Cell stage when crossed over 4-Cell stage (Figure 2C; Supplementary Figure S2), and these metabolites also enriched in glycometabolism-related sets such as fructose and mannose degradation and galactose metabolism (Figure 2D). Notably, fatty acids-related sets, including mitochondrial beta-oxidation of long chain saturated fatty acids, beta oxidation of very long chain fatty acids, oxidation of branched chain fatty acids and mitochondrial beta-oxidation of short chain saturated fatty acids, suggesting the lipid metabolism were enhanced at 8-Cell stage (Figure 2D). These data indicated the glycometabolism is necessary for each early cleavage stages and the embryo development.

### 3.3 Glucose promoted embryo development *in vitro*

**FIGURE 3:**
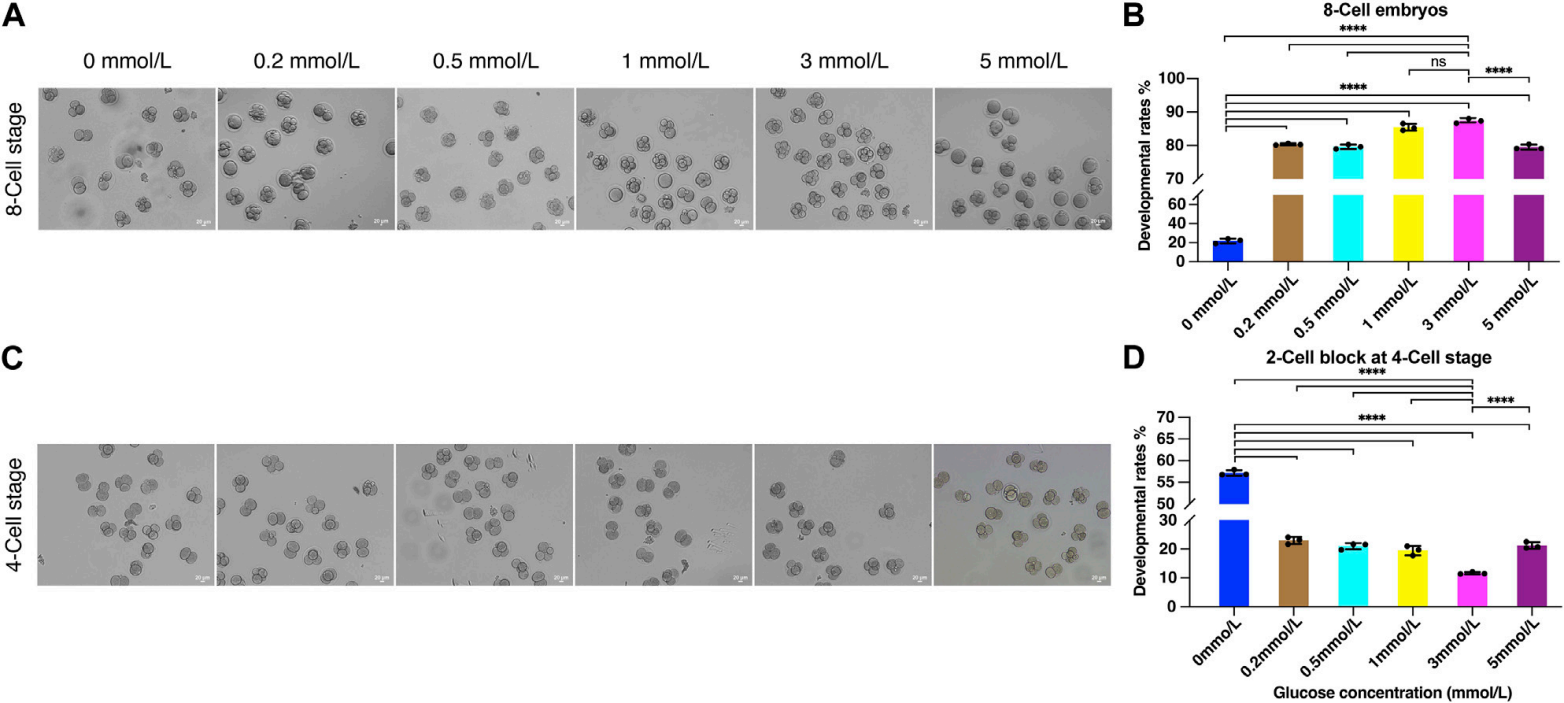
The effect of glucose on embryo development. **(A)** The embryo morphology cultured by different glucose concentrations at the 8-Cell stage; **(B)** The 8-Cell embryo rates of culture medium with different glucose concentrations; **(C)** The 2-Cell block embryos cultured by different glucose concentrations at the 4-Cell stage; **(D)** The 2-Cell block rates of culture medium with different glucose concentrations (**** represent for *p* < 0.0001).

To investigate the effect of glycometabolism on embryo development, and optimize the culture microenvironment to improve the embryo development potential, we examined the 8-Cell rate using modified KSOM culture medium with 0, 0.2, 0.5, 1, 3, and 5 mmol/L glucose. After fertilization, the fertilized eggs were cultured in HTF medium for 8 h and then transferred to KSOM containing different concentrations of glucose. After 72 h of culture, 8-cell embryos were counted (Figure 3A). 8-Cell rates were calculated compared to the number of zygotes in each group.

Remarkably, the inclusion of glucose in the culture medium could significantly improve embryo development. The 8-Cell rate was only 9.95% when cultured without glucose, while 0.2 mmol/L glucose sharply increased the 8-Cell rate to 80.35%, and the development rates were increased in a glucose concentration-dependent manner with 0.5 and 1 mmol/L, the 8-Cell rates were 79.61% and 85.46%, and achieve 87.33% with 3 mmol/L, which was significantly increased than other groups except 1 mmol/L, and that was 8.8 times compare to the rates cultured in 0 mmol/L glucose media (Figure 3B), suggesting the determined role of external glucose in embryo development microenvironment. However, the higher concentration at 5 mmol/L decreased the developmental rate to 79.52% (Figure 3B), suggesting the inhibition of development at high glucose concentration.

Notably, we observed substantial numbers of embryos were blocked at the 2-Cell stage when cultured without different concentrations of glucose (Figure 3C). Accordingly, we estimated the 2-Cell block rates at the 4-Cell stage, the data demonstrated the 2-Cell block rate was 57.13% when cultured without glucose, and significantly decreased to 11.63% when cultured in 3 mmol/L media (Figure 3D), suggesting the addition of glucose was helpful for embryos to overcome the 2-Cell block.

### 3.4 Glucose increased lipid synthesis at 2-cell stage

**FIGURE 4:**
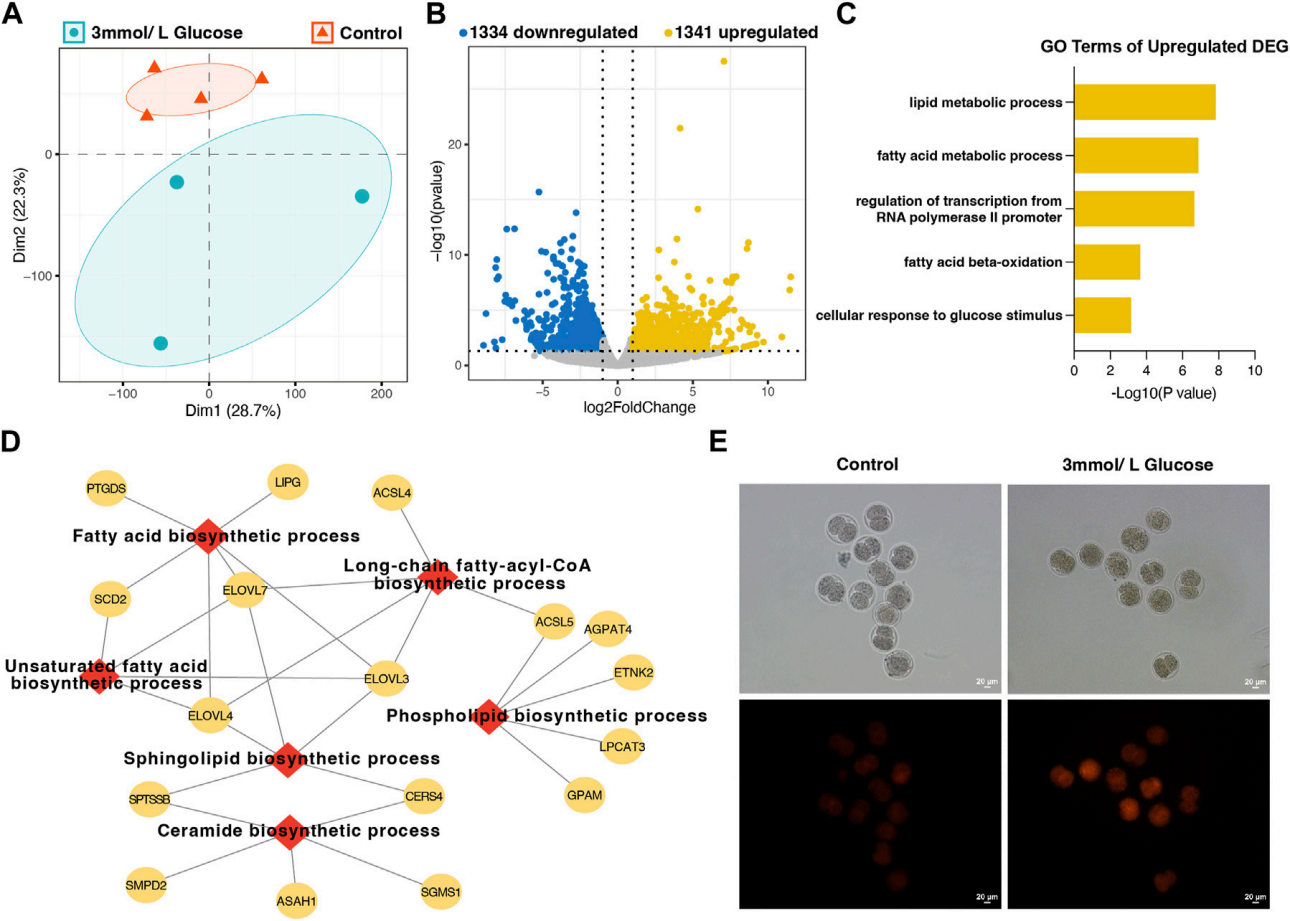
The transcriptome analysis and lipid detection of embryos cultured in different glucose concentrations at the 2-Cell stage. **(A)** The PCA plot of embryos cultured in control KSOM media and 3 mmol/L glucose media by the RNA-seq data; **(B)** The differentially expressed genes of embryos between the control group and 3 mmol/L glucose media group; **(C)** The GO enrichment analysis of upregulated genes in 3 mmol/L glucose media group; **(D)** The sub-enrichment analysis of 76 lipid metabolic process genes; **(E)** the Nile red staining of embryos in the control group and 3 mmol/L glucose media group.

To further explore the effects of glucose on improving the cleavage rate by reducing 2-Cell block, we detected the transcriptome of 2-Cell embryos cultured in different medium. PCA analysis showed the addition of 3 mmol/L glucose could obviously distinguished the embryos from which cultured in control KSOM media (with 0.2 mmol/L glucose), suggesting the increase of glucose concentration significantly affected the gene expression profiling (Figure 4A). Moreover, we identified 2,675 differently expressed genes (DEGs) between 3 mmol/L glucose group and control group, including 1,341 upregulated and 1,334 downregulated DEGs (Figure 4B). Gene enrichment analysis found the upregulated DEGs were mainly involved in lipid-related metabolic process, regulation of transcription from RNA polymerase II promoter as well as cellular response to glucose stimulus (Figure 4C), while the downregulated DEGs were enriched in RNA-related processing and negative regulation of transcription from RNA polymerase II promoter (Supplementary Figure S3), suggesting the increase of glucose may enhance the lipid metabolism and affect the transcription. We further analysis the 76 genes involved in the most significant GO term lipid metabolic process, and found the biosynthesis of various lipids is significantly enhanced, including fatty acid, ceramide, sphingolipid, phospholipid as well as long-chain fatty-acyl-CoA (Figure 4D), indicating the glucose increase the lipid biosynthesis at 2-Cell stage. Fluorescence staining confirmed that the content of lipid in embryos cultured in 3 mmol/L glucose KSOM media was significant increase than the control embryos (Figure 4E). These data showed the promotional effect of glucose on overcoming the 2-Cell stage block was mainly by enhanced the lipid synthesis.

## 4 Discussion

Although the critical role of glucose in metabolism has been widely studied, the effect of added glucose in culture media for early embryo cleavage is still controversial (Chronopoulou and Harper, 2015). In this study, we first determined the metabolic characteristics of the culture medium at each cleavage stage, we found 2-Cell stage mainly involved the glutathione metabolism and pentose phosphate pathway, and the 8-Cell stage was enriched in glycometabolism related set, while the 4-Cell stage is a transition state. Sliding window analysis further confirmed different forms of metabolites in glycometabolism in the culture medium at each stage. Additionally, we found the developmental potential of embryos is dose-dependent on glucose in the culture media until 3 mmol/L, while removing glucose mainly led to a 2-Cell block. RNA-seq data revealed that additional glucose may enhance lipid biosynthesis through the pentose phosphate pathway at the 2-Cell stage. These data were helpful for a better understanding of the metabolic features and effect of glucose during early development.

Culture medium is essential for the embryo development *in vitro*, various medium with different compositions, such as glucose, pyruvate, lactate, and amino acids, significantly affect the embryonic development competence (Morbeck et al., 2017). Glucose is the key sources of energy metabolism, the main controversy over the role of glucose in early embryonic development is whether it is beneficial or harmful. Previously study found glucose in culture medium, even at low concentration, inhibit the embryo development and eliminate glucose was benefit for overcoming the 2-cell block (Schini and Bavister, 1988; Chatot et al., 1989; Lawitts and Biggers, 1991), while others showed glucose is necessary for cleavage (Ludwig et al., 2001), but the concentration is determined for the embryo, for instance, the 5.5 mmol/L were found impaired the embryo quality (Coates et al., 1999), while 5.55 mmol/L concentration led to higher polyploid fertilization and affected cleavage rate in human (Michaeli et al., 2011). Other studies have suggested that the beneficial effects found with low concentrations of glucose on embryonic development have been overstated. Meanwhile, it is reported that glucose had no impact on embryonic development, embryos could develop to the blastocyst stage without glucose (Biggers and McGinnis, 2001). In this study we have proven glycometabolism exsits at each stage of cleavage, while the addition of glucose, from 0.2 to 5 mmol/L, is critical for overcoming the 2-Cell block and significantly improve the development rate compared to the absence of glucose in KSOM media, and the beneficial effects depend on the increase of the addition of glucose until 3 mmol/L, which reached the peak value of 87.33% at 8-Cell rate and 85.56% at blastocyte rate. Higher concentration, for instance 5 mmol/L, decrease the developmental rates to the level of 0.5 mmol/L. Thus, we confirmed although the absence of glucose in KSOM media could support the embryonic cleavage at a low level, the presence of glucose greatly increased the rate for 8.8 time, especially in 3 mmol/L.

Glucose metabolism mainly involves glycolysis, pentose phosphate pathway and hex osamine biosynthetic pathway (Ghosh et al., 2023). Glycolysis of glucose and glycogen was found at a low level until the morula stage across species, early activation of glycolysis may reduce developmental competence (Gardner and Lane, 1996; Dutta and Sinha, 2017; Sharpley et al., 2021), subsequently, the first cell fate specification during the morula and blastocyst stage is controlled by glucose (Chi et al., 2020). Therefore the EDTA was added to culture media to reduce glycolytic by inhibiting the activity of the glycolytic enzyme 3-phosphoglycerate kinase (3-PGK) at cleavage stages (Lane and Gardner, 2001). However, the total elimination of glucose from culture media significantly reduced the development rate, and embryos were mainly blocked at the 2-Cell stage, suggesting the importance of external glucose at cleavage stages, and the internal glucose or glycogen only is not enough for embryo cleavage especially overcoming the 2-Cell block, which is the main challenge for the embryo development *in vitro* (Goddard and Pratt, 1983). Controversially, Biggers found that glucose-free media could support zygote development to blastocyst (Biggers and McGinnis, 2001). Thus, the effects of glucose on embryonic development and its mechanisms still need to be further studied.

In most species, the maternal fatty acids, amino acids, pyruvate and lactate are used as the main carbon sources at the beginning of embryogenesis (Dutta and Sinha, 2017; Sharpley et al., 2021; Ghosh et al., 2023), while low-level pyruvate was found did not alter the ATP contents at 2-Cell stage (Zhang et al., 2021), indicating the substantial role of lipid and amino acids at early stage. It was reported that embryos rely on oxidative phosphorylation and the breakdown of fatty acids and amino acids as energy sources during early development (Ghosh et al., 2023). We found the addition of glucose remarkably improve the development through overcoming the 2-Cell block, and this beneficial effect may be caused by the increasing of the lipid metabolism related gene expression and enhance of lipid biosynthesis at 2-Cell stage. Meanwhile, the characteristic metabolites in 2-Cell stage media were enriched in pentose phosphate pathway, these data demonstrated the added glucose may be used in the PPP and the generated NAPDH was used for the lipid biosynthesis. Furthermore, we also noted that the sorbitol was increased in 8-Cell stage, suggesting the glucose may be used for the polyol pathway at this stage, which also depended on the NAPDH generated in PPP. The mechanism that the increased lipid at 2-Cell stage and the activated PPP or polyol pathway for the development competence need further investigation.

## 5 Conclusion

In conclusion, we found the glucose metabolism presented at each cleavage stage, the addition of glucose in the culture media is critical for embryo development, which may helpful for the overcoming of the 2-Cell block through increasing lipid biosynthesis. Considering the metabolites represent the result of the biological process, metabolites during the cleavage are the ideal diagnostic indicator that could be used to evolute the embryonic developmental potential. Thus, the determination of metabolic characteristics in the culture medium at each cleavage stage and the effect of glucose on the development could help the optimization of IVF culture conditions, as well as the non-invasive estimation of embryo quality using the culture media.

## Data Availability

The datasets presented in this study can be found in online repositories. The names of the repository/repositories and accession number(s) can be found below: OMIX, China National Center for Bioinformation/Beijing Institute of Genomics, Chinese Academy of Sciences (https://ngdc.cncb.ac.cn/omix), Accession no. OMIX004422.
