# Supplementary figures and images for: Glucose promoting the early embryonic development by increasing the lipid synthesis at 2-cell stage
Source: Front Cell Dev Biol. 2023 Jul 18;11:1208501. doi: 10.3389/fcell.2023.1208501 (PMC10392834; doi:10.3389/fcell.2023.1208501)

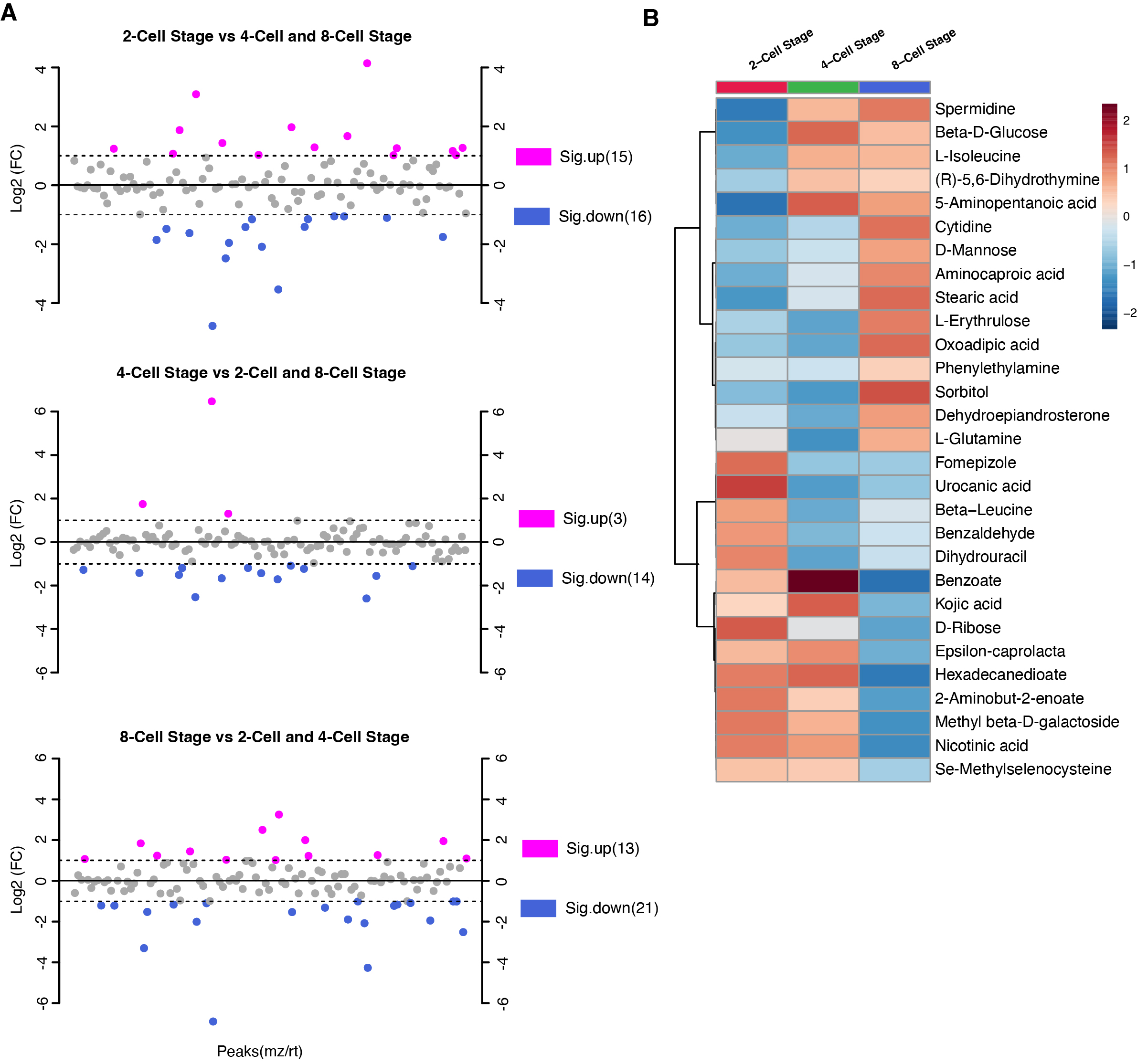

Supplement: Supplementary file 1 [file Image1.JPEG]

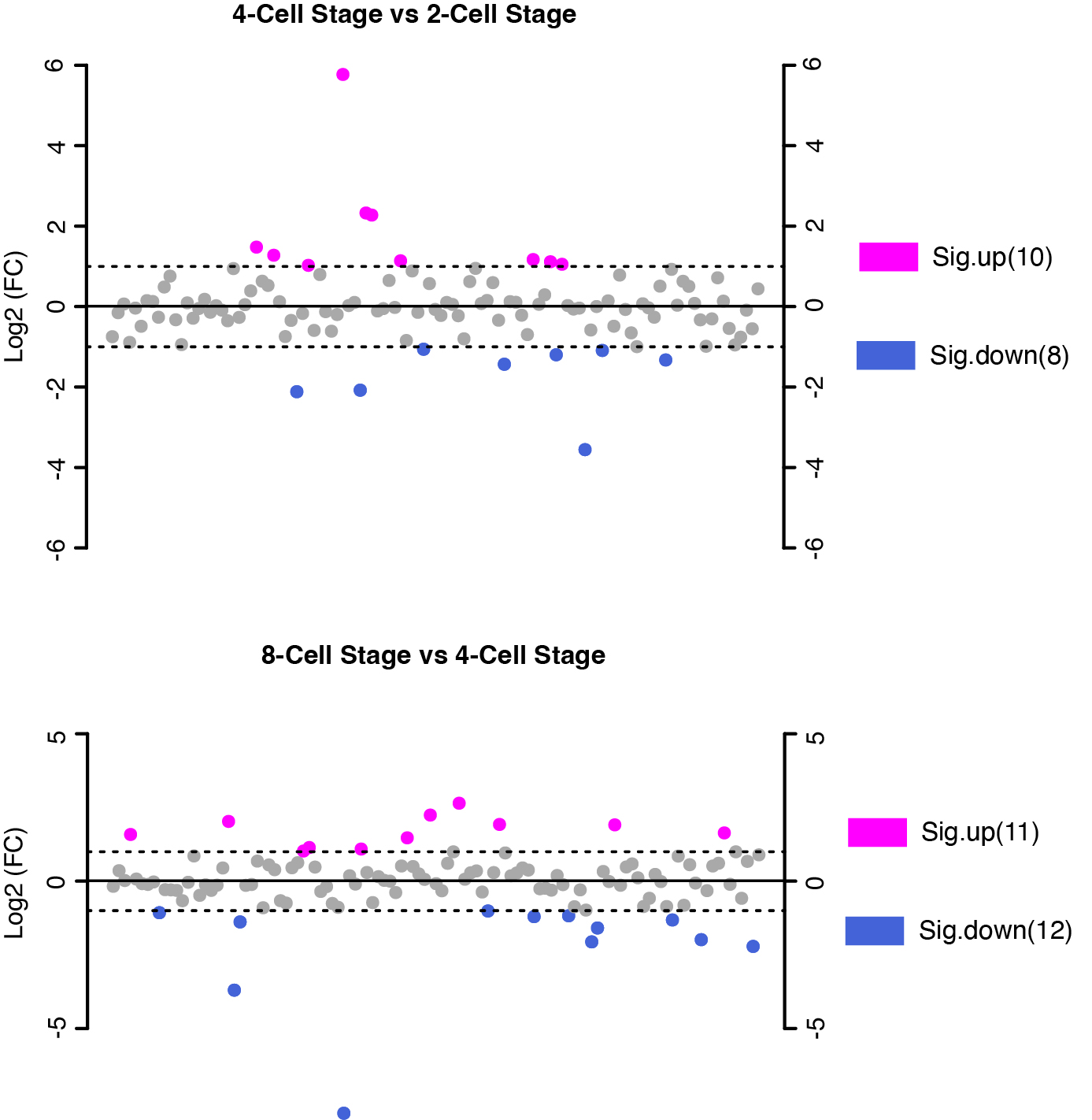

Supplement: Supplementary file 2 [file Image2.JPEG]
